# EARLYDRAIN- outcome after early lumbar CSF-drainage in aneurysmal subarachnoid hemorrhage: study protocol for a randomized controlled trial

**DOI:** 10.1186/1745-6215-12-203

**Published:** 2011-09-14

**Authors:** Jürgen Bardutzky, Jens Witsch, Eric Jüttler, Stefan Schwab, Peter Vajkoczy, Stefan Wolf

**Affiliations:** 1 Department of Neurology, Albert-Ludwigs-University Freiburg, Breisacherstr. 64, 79106 Freiburg, Germany; 2 Charité Center for Stroke Research Berlin (CSB), Campus Virchow Klinikum, Augustenburger Platz 1, 13353 Berlin, Germany; 3 Department of Neurology, Charité Campus Virchow Klinikum, Augustenburger Platz 1, 13353 Berlin, Germany; 4 Department of Neurology, Friedrich-Alexander-Universität Erlangen, Schwabachanlage 6, 91054 Erlangen, Germany; 5 Department of Neurosurgery, Charité Campus Virchow Klinikum, Augustenburger Platz 1, 13353 Berlin, Germany

## Abstract

**Background:**

Aneurysmal subarachnoid hemorrhage (SAH) may be complicated by delayed cerebral ischemia, which is a major cause of unfavorable clinical outcome and death in SAH-patients. Delayed cerebral ischemia is presumably related to the development of vasospasm triggered by the presence of blood in the basal cisterns. To date, oral application of the calcium antagonist nimodipine is the only prophylactic treatment for vasospasm recognized under international guidelines.

In retrospective trials lumbar drainage of cerebrospinal fluid has been shown to be a safe and feasible measure to remove the blood from the basal cisterns and decrease the incidence of delayed cerebral ischemia and vasospasm in the respective study populations. However, the efficacy of lumbar drainage has not been evaluated prospectively in a randomized controlled trial yet.

**Methods/Design:**

This is a protocol for a 2-arm randomized controlled trial to compare an intervention group receiving early continuous lumbar CSF-drainage and standard neurointensive care to a control group receiving standard neurointensive care only. Adults suffering from a first aneurysmal subarachnoid hemorrhage whose aneurysm has been secured by means of coiling or clipping are eligible for trial participation. The effect of early CSF drainage (starting < 72 h after securing the aneurysm) will be measured in the following ways: the primary endpoint will be disability after 6 months, assessed by a blinded investigator during a personal visit or standardized telephone interview using the modified Rankin Scale. Secondary endpoints include mortality after 6 months, angiographic vasospasm, transcranial Doppler sonography (TCD) mean flow velocity in both middle cerebral arteries and rate of shunt insertion at 6 months after hospital discharge.

**Discussion:**

Here, we present the study design of a multicenter prospective randomized controlled trial to investigate whether early application of a lumbar drainage improves clinical outcome after aneurysmal subarachnoid hemorrhage.

**Trial registration:**

www.clinicaltrials.gov Identifier: NCT01258257

## Background

Non-traumatic subarachnoid hemorrhage (SAH) is a major cause of stroke accounting for approximately 1-7% of cases. In 80% of SAH-cases the source of bleeding is a ruptured cerebral aneurysm [1,2]. Important for a patient's prognosis is the severity of the initial bleeding and complications associated with the presence of blood in the subarachnoid space. Once the aneurysmal SAH has occurred patients are predominantly threatened by two distinct problems in the acute phase. First, they may experience a further, often more severe, hemorrhage, and second, they may suffer delayed neurologic deterioration (DND) caused by delayed cerebral ischemia (DCI). The consequences of DCI may either be transient or may result in cerebral infarction with persistent neurologic disability or death.

The first problem, aneurysmal re-bleeding, is solved through rapid cerebrovascular imaging and subsequent treatment of the ruptured aneurysm, thus preventing recurrent hemorrhage. Aneurysm treatment may be performed either via craniotomy and surgical clipping of the aneurysm or using endovascular techniques by occluding the aneurysm with small platinum coils.

The second problem, DCI, is more difficult to recognize and to handle. Patients after aneurysmal SAH experience DND with an incidence of 30 to 60% [3]. It may be caused by hydrocephalus, cerebral edema, fevers, seizures, electrolyte abnormalities, and DCI. Strongly associated with DCI/cerebral infarction is a constringency reaction of the vessels supplying the brain with blood, called vasospasm [4]. The pathomechanism leading to vasospastic vessel constriction is not completely understood [5] and the quantitative relevance of vasospasm for the development of DCI is less clear than previously assumed [6].

Clinical signs of DND accompanying radiographic vasospasm are variable, depending on the affected blood vessels including alteration of mental status, aphasia, hemiparesis, or any other focal neurologic deficit. Often the consequences of this condition may include permanent neurologic deficits and death due to infarction and subsequent herniation of the brain. DCI, DND and vasospasm may be causatively interlinked, but also be independently present from each other. Vasospasm may be asymptomatic without clinically apparent deterioration of the patient's condition or external circumstances, such as deep sedation, may prevent clinical detection of a deterioration caused by vasospasm.

As DND is unspecific concerning its etiology, clinical judgment, therefore, is unreliable for the prediction and recognition of vasospasm. Thus digital subtraction angiography is the procedure of choice for the detection of vasospasm. Vasospasm may be present in the proximal vessels, the distal branches of the vasculature, or both.

Currently the only measure recognized for the prevention of DCI is the prophylactic application of the calcium channel blocker nimodipine [7]. Newer approaches, to date not included in official guidelines but pursued in several centers, include medication with statins and magnesium [8].

One hypothesis claims that the likelihood of angiographic vasospasm to occur is related to the amount of blood in the basal cisterns. According to this consideration, one prophylactic strategy is to remove as much of this blood as early as possible. If clipping of the aneurysm is performed this can be achieved intraoperatively by opening the terminal lamina and irrigating the blood from the basal cisterns. Albeit promising, studies addressing the efficacy of this measure show inconclusive results [9]. This approach is not feasible if the aneurysm is secured using an endovascular approach.

Excess removal of cerebral spinal fluid (CSF) via an external ventricular drain fails to prevent vasospasm and may lead to a higher incidence of posthemorrhagic shunt dependency [10,11]. Supposedly this is because after aneurysmal SAH, the blood settles and clots in the basal cisterns and therefore only CSF, being more lightweight, is removed via the ventricular drain.

Application of a lumbar drain has been proposed as an alternative approach to address clotting of the blood in the basal cisterns. In two retrospective studies in patients after aneurysmal SAH, the safety of this approach was shown [12,13]. One of these studies addressed the radiologic and clinical outcome after surgical clipping [12], while the other addressed the outcome after endovascular coiling [13]. Both studies led to a markedly diminished incidence of angiographic vasospasm and improvement in clinical outcome measured by the Glasgow Outcome Scale (GOS). Therefore a prospective study addressing the efficacy of this novel approach is warranted and currently being conducted (EARLYDRAIN).

The aim of the EARLYDRAIN study is to examine the efficacy of application of lumbar drainage in patients with acute subarachnoid hemorrhage from a ruptured cerebral aneurysm. The hypothesis is that early application of a lumbar drain after aneurysmal SAH leads to an improved outcome at six months after the hemorrhage, measured by the modified Rankin score. Furthermore, it is hypothesized that this postulated clinical effect will be due to a diminished incidence of cerebral vasospasm and delayed cerebral ischemia. Therefore the incidence of angiographic vasospasm and the development of new infarctions shown on CCT at discharge of the patient will be among the secondary endpoints of the present study.

## Methods

### Study design

The present study is in compliance with the Helsinki Declaration. Ethical approval was obtained by the ethical committee of the medical faculty of the Friedrich-Alexander-University Erlangen-Nürnberg (reference number: 4171).

The EARLYDRAIN study is a 2-arm randomized controlled trial to compare an intervention group receiving early continuous lumbar CSF-drainage and standard neurointensive care to a control group receiving standard neurointensive care only. It is conducted by a German national study group consisting of neurosurgical centers treating at least 30 patients with aneurysmal subarachnoid hemorrhage per year. Data management and monitoring will be performed by the Center for Stroke Research Berlin (CSB) at Charité University Medicine, Berlin, Germany.

Patients suffering from an aneurysmal SAH and completed elimination of the causative aneurysm are being recruited for this study. The choice of the method of aneurysm treatment is at the discretion of the neurovascular team taking care of a patient and not specified by the study protocol. All medical treatment is performed according to local guidelines and standard operating procedures.

### Subject Inclusion criteria

• Age: 18 years or older

• First aneurysmal SAH

• Pre-morbid modified Rankin Scale score 0 ("no symptoms at all") or 1 ("no significant disability despite symptoms")

• Aneurysm treatment performed during the first 48 hours after the initial hemorrhage.

• Informed consent by the patient or his/her legal representative. In case neither the patient is capable of giving informed consent nor a legal representative is available, informed consent can be given by an independent physician neither involved in the patient's treatment nor in conducting the trial.

### Subject Exclusion criteria

• Subarachnoid hemorrhage of other than aneurysmal origin

• No hemorrhage visible on initial CCT-scan (Fisher Grade I)

• Pregnancy

• Concurrent participation in another interventional trial (participation in an observational trial is not an exclusion criteria)

• Life expectancy less than 1 year for other reasons than the current SAH

• Other concomitant severe disease that would confound with treatment

• Other clear contraindication for treatment with a lumbar drain (e.g. absent or compressed basal cisterns on the admission CCT)

### Interventions

**Figure 1:**
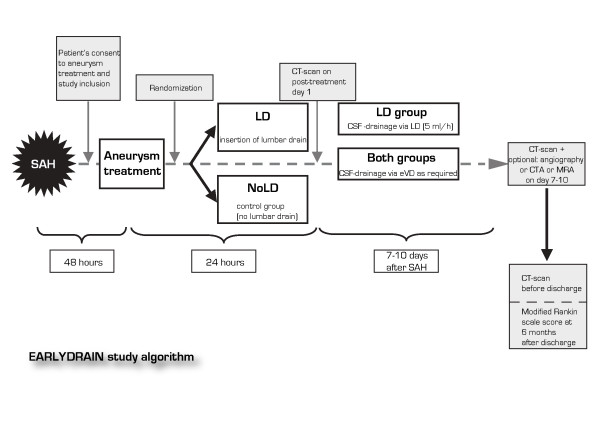
**EARLYDRAIN study algorithm**. The EARLYDRAIN study algorithm showing the course of events after the initial aneurysmal subarachnoid hemorrhage (SAH) and the subsequent surgical or interventional aneurysm treatment. The study includes two groups, a treatment group receiving lumbar CSF-drainage (LD) and a control group receiving no lumbar drainage according to protocol (NoLD). The timing of the patient's consent to study participation, randomization, cranial imaging and assessment of clinical outcome is indicated by the shaded boxes. Imaging on day 7 to 10 is scheduled according to local guidelines. If a local center performs no routine cerebrovascular imaging for vasospasm screening in patients without clinical suspicion, it may be omitted.

In order not to provoke premature rupture of the aneurysm due to accidental drainage, randomization to the study and eventual placement of a lumbar drain takes place after securing the aneurysm by the preferred method of choice (Figure 1). Every patient in the lumbar drainage group (LD-group) receives a lumbar drain during anesthesia required for the aneurysm treatment. Insertion of a lumbar drain into the subarachnoid space is conducted in standard fully sterile technique. This is to be performed before anticoagulation or anti-platelet therapy is initiated, which sometimes is warranted after endovascular coiling. A post-procedural CCT scan of the brain is performed within 24 hours after aneurysm treatment. In case of neurological worsening after the procedure it is strongly recommended to perform the follow-up CCT-scan as soon as possible.

In patients in the LD-group, CSF drainage via the lumbar drain is started slowly and steadily at a rate of approximately 5 ml per hour after the post-interventional CCT. This leads to a planned daily CSF-drainage of 120 ml per day through the lumbar route. Patients in both groups may receive additional CSF drainage via a ventricular device. The amount of CSF drained via the ventricular route is determined according to clinical requirement and not specified by the study protocol.

In order to enhance accuracy of the amount of CSF drained, regular drainage control every other hour and stopping in case of excess drainage is strongly recommended by the principal investigators. In case of neurological decline suspiciously related to the lumbar drainage, the drain must be closed immediately. Drainage may be gradually restarted after 12 to 24 hours, after performing a CCT scan.

If the post-procedural CCT or any other follow-up CCT scan shows compressed basal cisterns or any signs of threatening herniation, lumbar CSF diversion in the LD-group must not be performed. It may still be feasible to carefully drain CSF via the lumbar route [14], but this is at the discretion of the local investigator and not recommended. In patients requiring sedation and mechanical ventilation, either due to neurological impairment or for other medical reasons, intracranial pressure monitoring is mandatory. This may be performed according to local policy either with parenchymal or ventricular devices. If the intracranial pressure exceeds 20 mmHg, further CSF drainage via lumbar route shall be interrupted until the ICP is below 20 mmHg again. Careful CSF-drainage via the lumbar route may still be feasible in case of high intracranial pressure [14], but again this is at the discretion of the local investigator. A method of detecting the safety of lumbar CSF diversion may be determining the gradient of lumbar versus intracranial CSF pressure [15]. Albeit promising, the Earlydrain investigators explicitly rate this approach preliminary and experimental. In case of doubt, lumbar CSF diversion must not be performed.

Further neuromonitoring with transcranial duplex sonography (TCD), electroencephalography (EEG), brain tissue oxygenation recordings, jugular bulb oxymetry, regional cerebral blood flow measurement, microdialysis or other methods is at the discretion of the center and according to its local guidelines. As far as possible, this data should be saved electronically for post-hoc analysis.

A CCT scan as well as conventional digital subtraction angiography (DSA), CT angiography or MR angiography for assessment of vasospasm in the larger vessels is routinely performed on day 7 to 10 after the initial hemorrhage, according to local guidelines. In case of the occurrence of a DND when vasospasm is assumed to be the cause, angiography may be performed at any time. If it is performed earlier than day 7 to 10 and the patient shows no clinical deterioration thereafter, the angiography on day 7 to 10 is omitted. Treatment of radiographically confirmed vasospasm is according to local guidelines and not specified in the Earlydrain study protocol. It may include augmentation of cerebral blood flow via hypertensive hypervolemia as well as endovascular balloon dilation or intraarterial infusion of vasodilators.

After cerebrovascular imaging on day 7 to 10 the lumbar drainage of CSF is stopped in the LD-group. If cerebrovascular imaging is carried out before day 7, lumbar drainage is stopped on day 8. It may be pursued on a clinical base, as required.

### Amount and duration of CSF drainage

Patients randomized to the lumbar drainage group shall receive a daily drainage of 120 ml CSF, or 5 ml per hour for seven days. If higher amounts of CSF need to be drained on clinical grounds as in patients with hydrocephalus, this is preferably performed via an external ventricular drain.

The drain is planned to remain in place until the control angiography on day 7 to 10 after the initial hemorrhage. The local investigator may decide to remove the drain earlier in patients fully mobilized without clinical necessity of CSF drainage. However, consecutive drainage should not be less than four days to achieve a valid study result. Lumbar CSF drainage may be prolonged beyond the control angiography on clinical requirement. The amount of CSF drainage may then be adjusted to clinical requirements and bears no further restriction.

Patients randomized to the control group should not receive a lumbar drain before the planned control angiography to be performed on day 7 to 10 after SAH. If the patient develops hydrocephalus, and no EVD was placed initially for CSF drainage, a lumbar drain may be installed at the discretion of the local investigator. These patients are analyzed in the intention-to-treat analysis, but are not suitable for the per-protocol analysis.

### Consent to study participation

Consent to study inclusion is sought after explanation and agreement to a specific aneurysm treatment. Thus, patients capable of consenting to the aneurysm treatment will be informed about the study details themselves and may or may not agree to participate. If a patient is incapable of consenting to the proposed treatment, the legal representative should be informed on the conditions of treatment choices and afterwards, on the details of the EARLYDRAIN study. A patient may be randomized if the legal representative gives informed consent to the study, based on the presumed will of the patient. If neither the patient is capable of giving informed consent nor a legal representative is available in due time, an independent physician not involved in the patient's treatment nor in the trial may be asked for study approval. The latter option reflects a distinct characteristic of German law and the local ethic committee may or may not permit this.

This option was introduced into the consent procedure because the aforementioned retrospective data on lumbar drainage for treatment of aneurysmal SAH suggests a potentially beneficial effect of the measure for the patient. Therefore it shall not be categorically withheld from patients who are not capable of deciding whether to participate in the study or not and who do not have a legal representative.

However, in these cases of deferred consent, a legal representative needs to be established as soon as possible, according to German law. From our experience this legal procedure requires a time period from proposal to establishment of a legal representative of up to 72 hours, thus requiring the consent of an independent physician upfront for study inclusion. As soon as a legal representative is available and/or the patient is capable again to consent to the study, he or she must be asked to give informed consent. If the patient or his/her legal representative refuses consent after inclusion by advice of an independent physician, the patient's further study participation is no longer possible. In this case, however, the patient or his/her legal representative is asked to give consent for evaluation of already acquired data.

The detailed explanation of the study to the patient, legal representative or independent physician has to be carried out using appropriate explanations and words depending on the previous medical knowledge of the respective person and her/his level of education. During the explanations the respective person will be asked on a regular basis if she/he understands the conveyed information and if any questions have arisen. In addition to these verbal explanations the patient/legal representative/independent physician will be given a leaflet containing the study details. After reading the leaflet the respective person will be given as much time as she/he demands for the decision on study participation.

### Randomization

Any patient meeting the inclusion criteria and not violating the exclusion criteria may participate in the EARLYDRAIN study and be randomized to either receive a lumbar drain or not, thus defining the two distinct groups LD and NoLD.

Randomization is performed via a dedicated internet site accessible for all local investigators of the participating trial centers http://www.randomizer.at. No stratification or minimisation is to be used. The security measures of the online randomization system "randomizer.at" include that 1. All transactions are logged, 2. The audit trail of the trial can be accessed and analysed any time by the trial monitoring committee. 3. Network traffic between the web-browser and the randomizer is encrypted using SSL (*Secure Sockets Layer*) with strong encryption.

### Sample size calculation

In the ISAT trial, the largest trial on the treatment of aneurysmal subarachnoid hemorrhage so far, the mortality at one year follow-up was 8.1% to 10.1% [16]. Given the data from both retrospective studies on lumbar drains after SAH, a reduction from 15% to 2.1% after coiling and from 5% to 3% after clipping was shown. Thus, both studies were way lower in their mortality rate and, therefore, their external validity may be questioned.

In the two retrospective trials, 167 [12] and 107 [13] patients were studied, respectively. The effect of lumbar drainage was a decrease of the incidence of "clinical vasospasm" by 34% [12] and 40% [13], respectively.

In the above-mentioned studies the term "clinical vasospasm" includes neurological deterioration not explainable by hemorrhage, cerebral edema, hydrocephalus, hyponatremia, drug toxicity, infection or seizures. No distinction is made between delayed cerebral ischemia (DCI) and vasospasm as potential causes of the clinical worsening.

The following statistical calculations are based on the assumption that the clearly defined subtype of "delayed neurological deficit" measured in the above-mentioned two retrospective trials is highly correlated with clinical outcome 6 months after SAH, which constitutes the primary endpoint of the EARLYDRAIN study.

For lack of previous studies assessing clinical outcome after lumbar drainage in SAH as a primary endpoint this assumption seemed justified.

To assess a decrease of the incidence of DND from 40% to 20% in a prospective clinical trial, 93 patients in each of the two study arms are required to gain a power of 85%, using an alpha error of 5%. To account for possible imbalances in the randomization procedure concerning severity of clinical and radiological grading of the SAH or the choice of treatment and to facilitate a preplanned analysis on the severity of the initial hemorrhage, the planned study size is to include and randomize altogether 300 patients. This results in a power of 85.2%, again, using an alpha error of 5%, to detect a decrease in the rate of severe disability on a dichotomized modified Rankin scale from 50% to 33%, which would be consistent with the effect size from the retrospective trials on a dichotomized GOS [12,13]. The EARLYDRAIN investigators are aware of other, more conservative calculations for the sample size of patient-centered outcome studies targeting vasospasm, indicating that there may be the necessity to include more than 5000 patients in a single trial [17]. The power calculations, as described above and based on the available retrospective data, do not substantiate numbers this large. Besides feasibility issues, clinical experience from the principal investigators considering the expected effort-benefit ratio does not warrant enlargement of the trial to detect a rather small difference between groups.

### Safety of lumbar drains after aneurysmal SAH

In the above-mentioned two retrospective studies, mortality was lower in the lumbar drainage group. Neither of the retrospective studies mentions procedural related complications for the lumbar drains [12,13]. In patients with increased intracranial pressure, careful lumbar drainage of CSF may be a possible treatment even in case of compressed basal cisterns [14]. A feasible strategy to enhance safety is determination of the lumbar-cranial pressure gradient and cessation of lumbar CSF diversion in patients with increasing pressure difference [15].

However, in patients presenting increased intracranial pressure or compressed basal cisterns on CCT-scan the risk associated with lumbar drainage is unclear according to the current state of medical knowledge. In unclear cases the investigator must refrain from the insertion of a lumbar drain.

### Outcome assessment

The primary endpoint is disability after 6 months, assessed by the modified Rankin Scale [18] dichotomized at a score of 0 to 2 versus 3 to 6 (6 = death). Assessment is performed by a blinded investigator of the local study center by personal visit. Alternatively, a telephone questionnaire is suitable for outcome assessment using the modified Rankin Scale [19]. Outcome assessment is planned to be done on the whole dataset as well as in preplanned stratified subsets (i.e. for example clinical SAH grade according to the Hunt&Hess scale 1-2 vs. 3-5 [20], CT grading according to Fisher I-III vs. IV [21]).

Secondary outcome criteria are:

• Mortality after 6 months

• mRS score after 6 months as continuous variable

• Angiographic vasospasm on day 7 to 10, as defined by a caliber reduction by 33% or more compared to the initial digital subtraction angiography

• Endovascular rescue therapy performed due to proven vasospasm, using balloon dilation of spastic vessels and/or arterial infusion of vasodilators

• Infarction (due to vasospasm) in the last CT scan before discharge

• Expression of clinical delayed neurological deficit after the aneurysmal SAH until discharge from acute care.

• Daily course of TCD mean flow velocity in both MCA at a depth of 50-60 mm

• Rate of death during the initial hospital treatment after the aneurysmal SAH.

• Rate of CSF shunt insertion during the first six months

• Presence of CSF infection during the first 14 days, as defined by modified CDC criteria for device-associated meningitis (treatment required on either positive culture, or elevated cell count, red cell/white cell ratio, increased lactate and/or decreased glucose) [22].

The following parameters will be recorded and used in predictor-/association models concerning primary and/or secondary outcome parameters:

• Gender

• Age

• Hunt&Hess grade on admission

• Time from symptom onset to admission

• Location of aneurysm

• Time from symptom onset to aneurysm treatment

• Treatment of aneurysm by clipping or coiling or both

• Time from symptom onset to randomization

• Time from symptom onset to treatment start (i.e. insertion of the lumbar drainage in the treatment arm)

• Time from admission to discharge

• Insertion of EVD (yes/no)

• Duration of EVD being in place

• Duration of lumbar drainage

• Amount of CSF drained by EVD [ml]

• Amount of CSF drained by lumbar drain [ml]

• Use of nimodipine (yes/no)

• Use of statins (yes/no)

• Use of Mg^2+ ^(yes/no)

• Transcranial Doppler ultrasound in both MCA at 50-60 mm depth, 1x daily (> 160 cm/s versus < 160 cm/s)

• Presence of CSF infection during hospital stay (yes/no)

#### Data Management and Monitoring Body

All data specified in the trial protocol will be documented in the patient's records and on standardised Case Report Forms (CRFs), available as original with two copies. The investigating physician is responsible for appropriate completion of the form. The (CEHRIS) of the Center for Stroke Research Berlin (CSB) is responsible for data base development, data acquisition via double entry, data storage, and validation. Data validation includes controls of completeness, consistence and plausibility of the data documented in the CRF using a query system between data management and investigating physician. After resolution of all queries concerning enrolled patients, the data bank is closed (end of the trial) and forwarded to the biometrician for the purpose of evaluation. After finalization of all evaluations the final report and all original CRFs are delivered to the principal investigator.

The trial is supervised and monitored by the Intensive Care Treatment of Stroke group (ICTOS) of the CSB including initiation and regular site visits, source data verification, and reports of adverse events. All data management and supervising procedures are performed according to Standard Operation Procedures (SOPs) of the CSB and in accordance to ICH-GCP Guidelines (E6) and the declaration of Helsinki.

### Adverse events (AE) and severe adverse events (SAE)

Apart from AE and SAE which may occur after the beginning of the trial (synchronous with the insertion of the LD) there are complications related to securing the aneurysm:

Surgery-related complications: Surgical treatment includes the known risks of surgical interventions.

Complications related to endovascular therapy: Endovascular therapy includes the risks known to be associated with it.

### Definition of adverse events and severe adverse events

The term "adverse event" (AE) describes any sign, symptom, syndrome or any disease 1. occurring newly in a trial participant after consent to the trial and 2. being of particular interest for the assessment of the disease or the security of the therapeutic concept. In this trial AEs include:

• Arterial or venous thrombosis,

• Complications related to insertion of a lumbar drainage,

• Any SAE

The term AE does not implicate a causal correlation with the participation in the trial. Surgical or endovascular interventions are not necessarily considered as AE but can be necessary for the therapy of an AE. AEs are divided in severe (SAE) and non-severe (AE) adverse events.

An SAE is any AE occurring during the trial that is related to:

• Death

• Any life-threatening condition,

• Re-hospitalisation or prolongation of hospitalisation,

• Long-term or severe restraint of the state of health, or

• Birth deformities.

### Documentation

Investigation of AEs is part of every assessment of the study participants. Any AE has to be documented in the CRF.

Every SAE has to be documented on a special documentation form and has to be reported within 24 hours after recording, but at least at the next working day, to the data monitoring center in Berlin.

### Statistical Analysis

All data are described according to their mean, median or frequency, as applicable. The dichotomized modified Rankin score as primary outcome variable is investigated in using univariate analysis. Multivariate logistic regression modeling is performed accordingly, adjusted for clinical grade, fisher grade, ventricular hemorrhage, parenchymal hemorrhage, gender, nimodipine or other concomitant medical treatment. Analysis is planned as intention-to-treat as well as per protocol, excluding the patients who were treated with amounts of CSF drainage via lumbar drain deviating from the specified 5 ml/h or which needed a lumbar drain when randomized to the No-LD group.

### Interim Analysis

An interim analysis after inclusion of 10 patients will address safety issues. This analysis focuses on the secondary endpoints and SAEs only, especially the rate of death during hospital stay. During the interim analysis, the recruitment for the EARLYDRAIN study is not stopped. The frequency of further safety analyses will be adjusted to the recommendations of the data and safety monitoring board (DSMB).

#### Data and Safety Monitoring Board (DSMB)

Safety aspects of the trial are supervised by the DSMB. The DSMB consists of an independent stroke physician, a neurosurgeon, and a neurointensivist, neither involved in the planning nor conduction of the trial nor participating in the trial. The DSMB independently elects a chairman. The DSMB is responsible for critical evaluation and suggestions for improvement of the trial protocol and supervision of the trial course. The DSMB has to be informed about the results of safety issues, especially the number of AEs and SAEs in each treatment group at least after every 10 patients having been enrolled, but at least every 6 months starting with the day of inclusion of the first patient, but also whenever the Steering Committee believes this to be necessary. Based on the results of safety aspects the DSMB will recommend to continue or stop the trial. The members of the DMSB confer personally or via telephone and report their recommendations to the Steering Committee.

#### Steering Committee

The steering committee consists of the neurosurgical (Stefan Wolf, principal investigator) and the neurological (Jürgen Bardutzky) project manager along with the directors of the two leading trial centers (Stefan Schwab and Peter Vajkoczy). The Steering Committee is responsible for planning of the trial including funding, development of the trial protocol in cooperation with the participating centers, design of patient's and legal representative's information and informed consent, approval of the trial protocol and informed consent including later amendments by legal authorities and ethics committees, selection, verification, and recruitment of potential trial centers, design of the CRF, organisation of a randomization system on a 24-hours/7-days basis including a trial-phone hotline. Based on the recommendations of the Data Safety and Monitoring Board (DSMB) the Steering Committee decides on preliminary termination of the trial. The Steering Committee can also stop the trial preliminarily, if advised so for other reasons by the DSMB. Furthermore the Steering Committee has to give consent to reports and publication of trial results.

## Discussion

Here we describe the design of a multi-center prospective randomized controlled trial to investigate whether early lumbar drainage improves clinical outcome after aneurysmal subarachnoid hemorrhage.

If one assumes that the primary hemorrhage usually occurring outside of the hospital is difficult to prevent because carriers of aneurysms are usually asymptomatic, then - apart from elimination of the aneurysm itself - delayed cerebral ischemia due to radiographically detectable vasospasm constitutes the most important aspect of aneurysmal SAH that causes substantial morbidity and mortality. The pathophysiological mechanisms underlying either of these entities and possibly influencing each other have not been understood sufficiently. Arterial narrowing as seen on angiography may be highly correlated with unfavourable clinical outcome but it is assumed that outcome-defining factors are more diverse [6]. To account for the complexity of factors causing early clinical deterioration after aneurysmal SAH the rather abstract term "delayed neurological deficit" has been created. However not only the interaction between these factors but also their influence on clinical long-term outcome remain speculative.

The present study is based on the belief that in the majority of cases DND is caused by angiographically detectable arterial narrowing and that the occurrence of DND, including DCI, and radiographic vasospasm are major factors for unfavorable outcome.

Since the development of nimodipine as a prophylactic agent against DND and DCI no new treatment strategies have been included in international guidelines. This emphasizes the necessity of new ways to approach prophylaxis and therapy of DCI.

The hypothesis that lumbar drainage may improve outcome after SAH was derived from the results of two previous retrospective investigations that have suggested a beneficial effect concerning the development of clinical deterioration [12,13]. However, the aforementioned studies used development of "clinical vasospasm" as primary endpoint whereas in the EARLYDRAIN study the primary endpoint is degree of disability after 6 months assessed in a prospective blinded manner using the mRS-score. Thus EARLYDRAIN focuses on clinical outcome, allowing direct conclusions about the benefit of early lumbar drainage in patients having experienced an aneurysmal SAH. Furthermore by choosing clinical outcome as the primary endpoint the authors of the present study tried to avoid ambiguity due to heterogenous believes concerning the etiology of DND and the role of radiographic vasospasm as an outcome-influencing factor or a mere epiphenomenon, respectively.

The focus on clinical outcome is also a feature that clearly distinguishes the EARLYDRAIN study from the LUMAS trial ("Lumbar drainage after subarachnoid hemorrhage", NCT00842049). This study has been completed in February 2011 and its results are awaited.

The LUMAS trial is a Phase II randomized clinical trial, the primary endpoint is the incidence of delayed ischemic neurologic deficits within three weeks after the initial hemorrhage. Clinical outcome according to the modified Rankin Scale score at 10 days and 6 months after the ictus are among the secondary outcome measures. The focus of LUMAS are efficacy of lumbar drainage after aneurysmal SAH with respect to the primary endpoint. The results of this trial will be studied carefully by the EARLYDRAIN-investigators with regard to efficacy and safety of the employed methods. Because of reverse, but comparable primary and secondary endpoints of the two studies their results offer the opportunity of a combined analysis.

## List of abbreviations

AE: Adverse event; SAE: Severe adverse events; SAH: Subarachnoid hemorrhage; LD: Lumbar drainage; No-LD: No lumbar drainage; CSF: cerebrospinal fluid; CCT: cranial computed tomography; MRI: Magnetic resonance imaging; TCD: transcranial duplex sonography; DSA: digital subtraction angiography; CRF: case report form; mRS: Modified Rankin scale; GOS: Glasgow outcome scale; EVD: External ventricular drain; DND: Delayed neurological deficit; DCI: Delayed cerebral ischemia; MCA: Middle cerebral artery; CSB: Center for stroke research Berlin; ICTOS: Intensive care treatment of stroke study group/Charité Berlin; DSMB: Data and safety monitoring board; LUMAS: "Lumbar drainage after subarachnoid hemorrhage"-study.

## Competing interests

The authors declare that they have no competing interests.

## Authors' contributions

JB and SW concepted and designed the protocol and are responsible for study coordination. SS and PV devised the EARLYDRAIN name and provided important intellectual content. JW drafted the final manuscript and provides study center support and coordination. EJ organizes study center coordination and provided important intellectual content. All authors approved the final manuscript prior to submission for publication.
